# Diagnosis and Treatment of Mucinous Appendiceal Neoplasm Presented as Acute Appendicitis

**DOI:** 10.1155/2016/2161952

**Published:** 2016-03-14

**Authors:** Ioannis Kehagias, Apollon Zygomalas, Georgios Markopoulos, Thanasis Papandreou, Pantelis Kraniotis

**Affiliations:** Department of General Surgery and Department of Radiology, University Hospital of Patras, 26500 Patras, Greece

## Abstract

Appendiceal mucocele is a rare cause of acute abdomen. Mucinous appendiceal neoplasms represent 0.2–0.7% of all appendix specimens. The aim of this study is to report a case of a mucinous appendiceal neoplasm presented as acute appendicitis, discussing the clinical and surgical approach in the emergency setting. A 72-year-old female patient was admitted to the emergency department with a clinical examination indicative of acute abdomen. The patient underwent abdominal computed tomography scan which revealed a cystic lesion in the right iliac fossa measuring 8.3 × 5.2 × 4.1 cm, with calcified walls, and a mean density indicative of high protein content. The patient was taken to the operating room and a right hemicolectomy was performed. The postoperative course was unremarkable. The histopathological examination revealed a low-grade mucinous appendiceal neoplasm with negative regional lymph nodes. Ultrasound and CT are useful in diagnosing appendiceal mucocele and synchronous cancers in the emergency setting. The initial operation should include appendectomy and resection of the appendicular mesenteric fat along with any fluid collection for cytologic examination. During urgent appendectomy it is important to consider every mucocele as malignant in order to avoid iatrogenic perforation causing pseudomyxoma peritonei. Although laparotomy is recommended, the laparoscopic approach is not contraindicated.

## 1. Introduction

Appendiceal mucoceles (AM) or mucinous neoplasms are rare lesions characterized by a distended and mucus-filled appendix. They represent 0.2–0.7% of all appendix specimens [1–5]. Historically, Rokitansky in 1842 was the first who described the appendiceal mucocele as a dilatation of the appendiceal lumen by an abnormal accumulation of mucus [6]. The appendix epithelium contains many goblet cells and thus the accumulation of mucus is a typical finding. Because of this mucus-producing epithelium, the most common epithelial tumors of the appendix are mucinous and begin as mucoceles [7]. Appendiceal mucoceles are historically classified into four histologic subgroups: (1) simple retention cysts, (2) mucosal hyperplasia, (3) mucinous cystadenoma, and (4) mucinous cystadenocarcinoma [1]. The clinical presentation is rather unspecific. Most of these AM are asymptomatic but can become symptomatic because of inflammation, presenting as an acute appendicitis or by causing nonspecific abdominal pain. In young patients assuming an acute appendicitis, the preoperative diagnosis is rare. In older patients a preoperative diagnosis by computed tomography (CT) scan is more probable, which can easily detect the AM or even pseudomyxoma peritonei [8]. The preoperative diagnosis of AM helps to avoid accidental iatrogenic perforation during surgery. This is very important because it can lead to pseudomyxoma peritonei, characterized by peritoneal dissemination with high morbidity and mortality rate [9].

The aim of this study is to report a case of giant appendiceal mucocele presented as acute appendicitis, discussing the clinical and surgical approach in the emergency setting.

## 2.  Case Presentation

A 72-year-old female patient was admitted to the emergency department complaining of severe pain on her right lower quadrate (RLQ) of the abdomen with duration of 12 hours. Fever of 38°C was also present. Her clinical examination was indicative of acute abdomen with a palpable mass in the right iliac fossa. Her past medical history included dyslipidemia, hypertension, chronic constipation, lower extremity varices, and a total left hip replacement. Standard laboratory examination showed mild leukocytosis with increase of neutrophil count.

**Figure 1:**
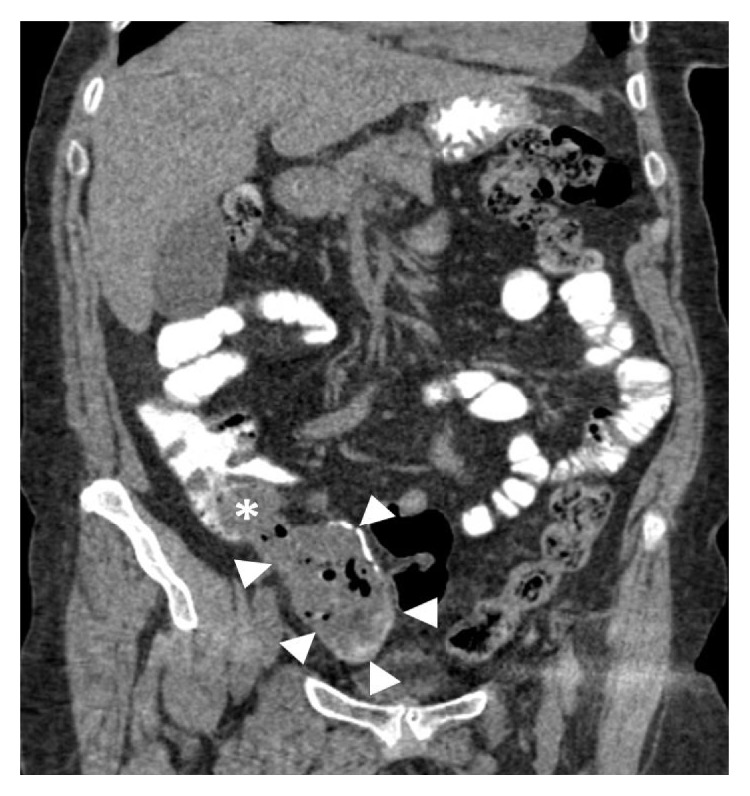
NECT-coronal MPR, showing the lesion (white arrowheads) in continuity with the cecum (asterisk).

The patient underwent a nonenhanced 16X multidetector abdominal CT scan, after oral administration of contrast solution (sodium amidotrizoate and meglumine amidotrizoate solution [Gastrografin®, Bayer Schering Pharma]). There was a cystic lesion in the right iliac fossa measuring 8.3 × 5.2 × 4.1 cm, contiguous to the cecum, consistent with a dilated appendix (Figure 1). The lesion had calcified walls. The mean density within the lesion was ~27 HU (due to high protein and/or mucinous content), with the presence of some hyperdense inner lines/foci, probably due to Gastrografin. There were also multiple air bubbles with air-fluid levels. Minimal fat stranding was evident around the lesion. There were no enlarged regional lymph nodes or free intraperitoneal air.

**Figure 2:**
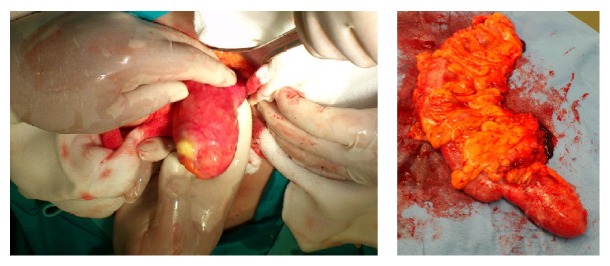
Intraoperative finding.

The patient was taken to the operating room after being given intravenous antibiotics (cefuroxime 1.5 gr and metronidazole 500 mg) and low-molecular-weight heparin. Taking into consideration the size of the lesion, the possibility of a malignant neoplasm and the prospective of an emergency right hemicolectomy in an elder patient, the open approach was decided. A midline incision was made and the peritoneal cavity was entered. An 8 × 5 × 4 cm mucocele of the appendix was found (Figure 2). There were no other significant findings. Frozen section was not available at the time of the operation. The age of the patient and the large appendiceal mass put a high suspicion of malignancy. In this context we decided to proceed with a right hemicolectomy. The gastrointestinal tract continuity was established by laterolateral stapled ileotransverse anastomosis. The patient's postoperative course was unremarkable, and she was discharged home on the seventh postoperative day. The histological examination revealed a low-grade mucinous appendiceal neoplasm with negative regional lymph nodes and without presence of mucinous peritoneal carcinomatosis. In addition two small tubular adenomas of the ascending colon with low-grade epithelial hyperplasia were revealed. No further surgical therapy was required. Oncologic consultation was recommended to the patient. Medical oncology did not recommend adjuvant chemotherapy. Follow-up with CT scans every six months and also CEA and CA19-9 tumor marker surveillance has been performed. One year postoperatively the patient is still alive.

## 3. Discussion

We presented the diagnostic and therapeutic approach in the emergency setting of a case of a giant appendiceal mucinous neoplasm presented as acute appendicitis in an elderly patient. Appendiceal mucinous neoplasms can be presented as acute appendicitis in only 8% to 14% of the cases [3, 10]. Furthermore, only 5–10% of cases of acute appendicitis occur in the elderly population [11]. Computed tomography scan is an important tool for the preoperative diagnosis in the emergency setting. However, the diagnosis of an appendiceal mucinous neoplasm is intraoperative and on histopathological examination.

The term appendiceal mucocele refers to a dilated appendix with increased intraluminal accumulation of mucus. Chronic obstruction of the appendix either by mucus or as a result of mucosal hyperplasia and benign or malignant neoplasms cause the appendiceal mucocele [1, 12]. Benign AM are more common with respect to the malignant variants and account for 63–84% of the cases. They are characterized by increase of the appendiceal diameter and epithelial villous adenomatous changes with epithelial atypia [1]. The malignant variants of AM are mucinous cystadenocarcinomas which represent 11–20% of the cases. They demonstrate severe luminal distension and glandular stromal invasion with or without peritoneal implants of epithelial cells [1, 12]. Mucocele of the appendix can also result from fecal impaction or polyps of the cecum which can obstruct the appendiceal ostium. Rare causes found in the literature are endometriosis and metastatic melanoma [13, 14].

A consensus for classification and pathologic reporting of mucinous appendiceal neoplasia was recently published [15]. It was agreed that the term “mucinous adenocarcinoma” should be reserved for infiltrative lesions. Furthermore, the term “cystadenoma” should no longer be recommended. Finally the terms “low-grade” and “high-grade” appendiceal neoplasm can be used for lesions without infiltrative invasion but with the corresponding low or high grade of cytologic atypia. Consensus was also achieved on the pathologic classification of pseudomyxoma peritonei (PMP) which was defined as the intraperitoneal accumulation of mucus due to mucinous neoplasia. Pseudomyxoma peritonei was classified into three categories: low grade, high grade, and high grade with signet ring cells. Low-grade and high-grade PMP are synonymous to disseminated peritoneal adenomucinosis and peritoneal mucinous carcinomatosis, respectively.

Recent reports showed a male predominance (5 : 2) [16]. However, AM are considered to occur more frequently in women [17]. In a retrospective study of 135 patients by Omari et al. 55% were females [10]. Mucoceles prevail in the 5th and 6th decades of life, though they may be diagnosed at any age [3]. Other tumors of the gastrointestinal tract, ovary, breast, and kidney can be associated with the presence of AM in up to one-third of the patients [10, 18]. Omari et al. recommend surveillance colonoscopy in patients with a diagnosis of AM, at least in those with diagnosis of appendiceal cystadenoma [10].

Ruiz-Tovar et al. reported 14% of their patients had an intraoperative diagnosis of appendicitis with AM [3]. Omari et al. in their retrospective study reported the clinical syndrome of acute appendicitis in 8% of the cases studied [10]. Other symptoms included abdominal pain, abdominal mass, weight loss, nausea or vomiting obstipation, and change in bowel habits. In the emergency setting AM can also be presented as intestinal strangulation, appendiceal intussusception, or generalised abdominal pain [5, 19, 20]. Approximately 30% of patients may present with perforated appendicitis or extravasation of mucus during surgery and this can result in pseudomyxoma peritonei [10]. Although both the benign and malignant variants of AM may cause pseudomyxoma peritonei, this is more frequent and with worse prognosis for malignant cases [3, 10, 21].

From those patients with perforation or extravasation, up to 83% may have a malignant mucocele [10]. A malignant AM may be present in 13% of patients without pseudomyxoma peritonei [10]. In a retrospective review study of Esquivel and Sugarbaker the most common initial symptom of patients with pseudomyxoma peritonei was appendicitis; nevertheless in none of these cases did the appendicitis occur as a first event of the dissemination [22].

Ultrasound and CT imaging studies are valuable for the detection of AM and can be easily performed in the emergency setting [16, 18, 23]. Ultrasound examination can detect AM with a sensitivity of 83% and a specificity of 92%, using 15 mm or more as threshold [24]. Although the lesion size is not associated with malignancy AM smaller than 2 cm are rarely malignant. Simple mucoceles have a mean diameter of 4.1 cm while cystadenomas have 8.1 cm [10]. Ultrasound may reveal a mass with fine echo spots and/or concentric, echogenic layers (“onion skin”), thought to be specific alteration [25, 26]. Computed tomography can be used in order to confirm the diagnosis and also allows for a better and precise study of the relation between the lesion and the neighbor organs [27]. In cases of acute appendicitis there may be an overlap with acute appendicitis without mucocele, though features suggestive of a coexisting mucocele include well-circumscribed cystic dilatation with low attenuation, mural calcification, and a luminal diameter greater than 1.3 cm [3, 8]. Curvilinear mural calcifications are very suggestive of mucocele and can be revealed in up to 50% of the cases [28–30]. Fine needle aspiration should be avoided to preserve the integrity of the cyst [27].

The optimal surgical approach for treating an appendiceal mucocele remains controversial. Traditionally neoplasms of the appendix more than 2 cm in diameter are managed by right colon resection. The rationale for this approach is the resection of occult lymph nodal metastases within the ileocolic lymphatic system [7]. At the time of appendectomy in the emergency setting, gross examination and the assessment of the size of the mucocele cannot reveal the malignancy of the lesion [31]. In these cases it is important to consider every mucocele of the appendix as malignant [7].

The laparoscopic approach has been described for the management of the appendiceal mucocele and is still recommended by some authors in selected patients [32, 33]. Single-port laparoscopic surgery for appendiceal mucoceles has also been reported to be safe and feasible [34]. However, González Moreno et al. suggest conversion to open appendectomy in case of mucocele revealed during laparoscopic appendectomy [35]. The open approach permits a safe and gentle surgical manipulation of the lesion. Furthermore port site recurrence after laparoscopic approach has been reported [35].

The initial operation should include appendectomy with en-block resection of the appendicular mesenteric fat and any fluid or mucus must be recovered for cytologic examination [7, 36]. Inside the appendicular mesentery and along the appendiceal artery approximately four to eight nodes are lying [7, 37]. These lymph nodes should be submitted for frozen section and if negative, right hemicolectomy is not indicated. Furthermore a positive margin on the base of the appendix can be managed by cecectomy alone in order to obtain a negative margin and thus save the ascending colon and the ileocecal valve function [7, 36]. Usually the initial surgery is urgent and the frozen section is not available; in that case a right hemicolectomy should not be performed since the malignant neoplasm is the cause of mucocele in only 10–20% of the cases [1, 7]. However, in case there is high suspicion for malignancy, the resection should be complemented with right hemicolectomy [36]. If a ruptured appendiceal mucocele is revealed intraoperatively, then the primary resection should be accompanied by removal of all gross implants [7, 35]. A complete abdominal exploration during the initial operation is indicated due to the occurrence of synchronous tumors and possible peritoneal seedlings. This approach is highly indicated when the surgery is performed with urgency and specific and accurate preoperative examinations have not been made [38].

After an initial urgent operation if the histological diagnosis reveals positive lymph nodes, adenocarcinoma of the intestine, mucinous adenocarcinoma, carcinoid or adenocarcinoid tumors larger than 2.0 cm, or high mitotic rate, a right hemicolectomy should be performed [39, 40]. Patients with perforated AM in the initial surgery but with negative lymph nodes or margins in the histological diagnosis should not be submitted for a right hemicolectomy as they present lower survival rates when compared to those who only had an appendectomy at the time of the primary surgery [41]. If the histological exam shows the presence of mucinous peritoneal carcinomatosis, then the patient will need cytoreductive surgery and hyperthermic intraperitoneal chemotherapy (HIPEC) with the prospective of a long-term survival [42, 43]. Low-grade tumors have the maximum survival benefit from these locoregional treatments [42]. However, only peritoneal carcinomatosis nodules between 2 mm and 5 mm can be adequately treated with intraperitoneal chemotherapy, even when combined with heat and thus cytoreductive surgery is essential [44]. Systemic chemotherapy before cytoreductive surgery and HIPEC may improve the prognosis in patients with peritoneal mucinous carcinomatosis [45].

In case of an appendiceal specimen with perforation and adenomucinosis, follow-up with CT scans every six months for five years and also CEA and CA19-9 tumor marker surveillance is recommended [7, 46]. If there is perforation of the AM with a diagnosis of mucinous adenocarcinoma a second-look surgery should be recommended. The timing for the second look should be at six months after the initial appendectomy. This selective second look should be used in order to prevent the rapid progression of a mucinous adenocarcinoma that may not be recognized by CT and tumor marker surveillance [42]. However in those patients with perforated AM the prognosis will be determined by chemotherapy and further cytoreduction surgery if neoplastic seeding exists and not by the type of surgical operation and thus appendectomy or right hemicolectomy [41, 47, 48]. Misdraji et al. reported no recurrence within a six-year follow-up for low-grade mucinous neoplasms, confined to the appendix, but only a 45% 5-year survival for the same low-grade tumor with extra-appendiceal spread [31]. Yakan et al. in their retrospective study on AM presented with acute abdomen or acute appendicitis reported no postoperative morbidity or mortality and an average postoperative length of hospital stay of 3.4 (2–7) days [5].

In conclusion appendiceal mucocele is a rare cause of acute abdomen. Ultrasound and CT are useful in diagnosing appendiceal mucocele and synchronous cancers in the emergency setting. However, the diagnosis is intraoperative and on histopathological examination. The initial operation should include appendectomy and resection of the appendicular mesenteric fat along with any fluid or mucus collection for cytologic examination. During urgent appendectomy it is important to consider every mucocele as malignant in order to avoid iatrogenic perforation causing pseudomyxoma peritonei. Although laparotomy is recommended, the laparoscopic approach is not contraindicated.
