# Corrigendum to “Diagnosis and Treatment of Mucinous Appendiceal Neoplasm Presented as Acute Appendicitis”

**DOI:** 10.1155/2016/3612014

**Published:** 2016-11-09

**Authors:** Ioannis Kehagias, Apollon Zygomalas, Georgios Markopoulos, Thanasis Papandreou, Pantelis Kraniotis

**Affiliations:** Department of General Surgery and Department of Radiology, University Hospital of Patras, 26500 Patras, Greece

In the article titled “Diagnosis and Treatment of Mucinous Appendiceal Neoplasm Presented as Acute Appendicitis” [1] reference [17] should replace reference [10]. As a result, the list of references from [10] to [17] will be as follows:

[10] L. Stocchi, B. G. Wolff, D. R. Larson, and J. R. Harrington, “Surgical treatment of appendiceal mucocele,”* Archives of Surgery*, vol. 138, no. 6, pp. 585–590, 2003.
[11] A. H. Omari, M. R. Khammash, G. R. Qasaimeh, A. K. Shammari, M. K. B. Yaseen, and S. K. Hammori, “Acute appendicitis in the elderly: risk factors for perforation,”* World Journal of Emergency Surgery*, vol. 9, article 6, 2014.
[12] A. J. Aho, R. Heinonen, and P. Lauren, “Benign and malignant mucocele of the appendix. Histological types and prognosis,”* Acta Chirurgica Scandinavica*, vol. 139, no. 4, pp. 392–400, 1973.
[13] A. A. Alduaij, M. B. Resnick, M. Kawata, and V. E. Pricolo, “Metastatic malignant melanoma presenting as an appendiceal mucocele,” * Journal of Oncology*, vol. 2011, Article ID 546570, 4 pages, 2011.
[14] D. K. Driman, D. E. Melega, G. A. Vilos, and E. A. Plewes, “Mucocele of the appendix secondary to endometriosis: report of two cases, one with localized pseudomyxoma peritonei,”* American Journal of Clinical Pathology*, vol. 113, no. 6, pp. 860–864, 2000.
[15] N. J. Carr, T. D. Cecil, F. Mohamed et al., “A consensus for classification and pathologic reporting of pseudomyxoma peritonei and associated appendiceal neoplasia: the results of the Peritoneal Surface Oncology Group International (PSOGI) modified delphi process,”* The American Journal of Surgical Pathology*, vol. 40, no. 1, pp. 14–26, 2016.
[16] K. Sasaki, H. Ishida, T. Komatsuda et al., “Appendiceal mucocele: sonographic findings,”* Abdominal Imaging*, vol. 28, no. 1, pp. 15–18, 2003.
[17] J. Misdraji, “Mucinous epithelial neoplasms of the appendix and pseudomyxoma peritonei,”* Modern Pathology*, vol. 28, supplement 1, pp. S67–S79, 2015.

As a result, the fourth and fifth paragraphs in the Discussion section should read as follows:

Recent reports showed a male predominance (5 : 2) [16]. However, AM are considered to occur more frequently in women [17]. In a retrospective study of 135 patients by Stocchi et al. 55% were females [10]. Mucoceles prevail in the 5th and 6th decades of life, though they may be diagnosed at any age [3]. Other tumors of the gastrointestinal tract, ovary, breast, and kidney can be associated with the presence of AM in up to one-third of the patients [10, 18]. Stocchi et al. recommend surveillance colonoscopy in patients with a diagnosis of AM, at least in those with diagnosis of appendiceal cystadenoma [10].

Ruiz-Tovar et al. reported 14% of their patients had an intraoperative diagnosis of appendicitis with AM [3]. Stocchi et al. in their retrospective study reported the clinical syndrome of acute appendicitis in 8% of the cases studied [10]. Other symptoms included abdominal pain, abdominal mass, weight loss, nausea or vomiting obstipation, and change in bowel habits. In the emergency setting AM can also be presented as intestinal strangulation, appendiceal intussusception, or generalised abdominal pain [5, 19, 20]. Approximately 30% of patients may present with perforated appendicitis or extravasation of mucus during surgery and this can result in pseudomyxoma peritonei [10]. Although both the benign and malignant variants of AM may cause pseudomyxoma peritonei, this is more frequent and with worse prognosis for malignant cases [3, 10, 21].
